# Effect of Cilostazol on Delayed Cerebral Infarction in Aneurysmal Subarachnoid Hemorrhage Using Explainable Predictive Modeling

**DOI:** 10.3390/bioengineering10070797

**Published:** 2023-07-03

**Authors:** Kwang Hyeon Kim, Byung-Jou Lee, Hae-Won Koo

**Affiliations:** Department of Neurosurgery, College of Medicine, Inje University Ilsan Paik Hospital, Goyang 10380, Republic of Korea; kh.kim@paik.ac.kr (K.H.K.); lbjguni@hanmail.net (B.-J.L.)

**Keywords:** delayed cerebral infarction (DCI), angiographic cerebral vasospasm (ACV), explainable artificial intelligence (XAI), cilostazol, nimodipine

## Abstract

The studies interpreting DCI, a complication of SAH, and identifying correlations are very limited. This study aimed to investigate the effect of cilostazol on ACV and DCI after coil embolization for ruptured aneurysms (n = 432). A multivariate analysis was performed and explainable artificial intelligence approaches were used to analyze the contribution of cilostazol as a risk factor on the development of ACV and DCI with respect to global and local interpretation. The cilonimo group was significantly lower than the nimo group in ACV (13.5% vs. 29.3; *p* = 0.003) and DCI (7.9% vs. 20.7%; *p* = 0.006), respectively. In a multivariate logistic regression, the odds ratio for DCI for the cilonimo group, female sex, and aneurysm size was 0.556 (95% confidence interval (CI), 0.351–0.879; *p* = 0.012), 3.713 (95% CI, 1.683–8.191; *p* = 0.001), and 1.106 (95% CI, 1.008–1.214; *p* = 0.034). The risk of a DCI occurrence was significantly increased with an aneurysm size greater than 10 mm (max 80%). The mean AUC of the XGBoost and logistic regression models was 0.94 ± 0.03 and 0.95 ± 0.04, respectively. Cilostazol treatment combined with nimodipine could decrease the prevalence of ACV (13.5%) and DCI (7.9%) in patients with aSAH.

## 1. Introduction

Aneurysmal subarachnoid hemorrhage (aSAH) is a catastrophic disease with very high mortality and morbidity, which accounts for 5% of all stroke cases and affects approximately 9 in 100,000 people annually worldwide [1]. The mortality rate for aSAH has been estimated to be 30%. The outcomes of aSAH depend on several factors, including the subarachnoid hemorrhage (SAH) volume, chronic disease of the patient, and incidence of complications.

Among aSAH complications, angiographic cerebral vasospasm (ACV), which commonly occurs 3–14 days after the onset of aSAH, is the leading cause of high morbidity and mortality in patients with ruptured aneurysms [2]. If the ACV is very severe, ischemic cerebral infarction may occur, which can significantly impact the patient’s prognosis. Although there have been many studies conducted to identify ways to reduce ACV, the fatality rate is still high (10–20%) [3,4]. ACV can be diagnosed based on neurological deterioration, transcranial Doppler ultrasonography, and radiologic angiography. The pathophysiology of ACV following aSAH is not yet fully understood. Because calcium channel blockers (CCB) can inhibit the constriction of vascular smooth muscle cells, nimodipine medication has been recommended as the first-line drug to prevent ACV [5,6,7,8]. Despite adequate nimodipine medication, many patients develop severe ACV and delayed cerebral infarction (DCI).

Cilostazol is an inhibitor of phosphodiesterase (PDE) III, which leads to an increase in intracellular cyclic adenosine monophosphate (cAMP) content and acts as an antiplatelet agent, peripheral vasodilatory agent, and neuroprotective agent [9]. The prevalence of ACV and DCI can be decreased in patients with aSAH when they are administered cilostazol [10,11,12]. However, there is still a lack of consensus among the related studies.

Recently, data analysis using artificial intelligence (AI), including machine learning (ML), from refined data to find hidden features has been reported to illustrate the related risk factors [13,14]. However, there are few examples of data on complications such as ACV and DCI in patients with aSAH. Additionally, while many existing AI and conventional research cases focus on the accuracy of outcome prediction based on a specific predictive model, there are still limitations in interpreting the correlation of factors contributing to the result [15,16,17]. Lee et al. formulated a decision rule employing logistic regression, incorporating clinical and laboratory data, to forecast the occurrence of DCI in patients diagnosed with aSAH. Nonetheless, the authors did not furnish specific details regarding individual interactions or correlations concerning aSAH outcomes [16]. Tanioka et al. employed a machine learning approach, specifically, the random forest model, to assess the significance of matricellular proteins and clinical data with DCI. Nevertheless, a notable limitation of their study was the inability to derive precise relationships with some numerical values for the individual effects of prognostic factors on patients diagnosed with aSAH [17].

Therefore, this study aims to investigate the effect of cilostazol on complications such as ACV or DCI using the explainable AI (XAI) modeling technique in patients with aSAH. In this empirical investigation, we employ a combined approach, integrating the conventional method and explainable artificial intelligence techniques, to comprehensively examine the significance of prognostic factors influencing the incidence of DCI and the prediction of their impact on individual patients.

## 2. Materials and Methods

### 2.1. Data Collection

This study was a retrospective single-center study of patients with aSAH. Electronic database searches were used to identify consecutive patients with aSAH. A total of 432 consecutive patients with aSAH who presented to our hospital between January 2011 and December 2020 were enrolled. The inclusion criteria included patients who (1) were more than 18 years old, (2) had undergone a brain radiologic exam that confirmed the vasospasm and infarction, (3) had undergone clipping or coiling, and (4) received cilostazol and nimodipine 24 h after admission for at least 14 days. Patients who died within 14 days after admission or received other antiplatelet agents than cilostazol were excluded. Out of the 432 patients with aSAH, 39 patients died within 7 days, and 41 patients received other antiplatelet agents. During admission, 6 patients received cilostazol for 5–7 days only because they developed a headache after taking the cilostazol. Additionally, 7 patients received short-term medication of nimodipine due to low blood pressure, and 18 patients did not undergo any postoperative radiologic evaluation, such as computed tomography angiography (CTA) or magnetic resonance angiography (MRA), during their admission.

Several basic demographic features and risk factors were assessed in the patients with aSAH, including age at admission, sex, hypertension (HTN), diabetes mellitus (DM), hyperlipidemia, smoking history, aneurysm size (longest diameter) and location, treatment method for a ruptured aneurysm, Glasgow Coma Scale (GCS) score on admission, Hunt–Hess grade and Fisher grade on admission, ACV, and DCI. HTN was defined based on a prior diagnosis and the intake of antihypertensive drugs.

ACV was defined as moderate stenosis of more than 50% on CTA, MRA, or DSA. DCI was defined as the development of a new infarction on CTA or diffusion MRA, a new focal or global neurological deterioration, or a decrease of 2 points on the GCS score that was not explained by other medical conditions [18]. DCI was evaluated 2–14 days after admission using the DCI definition [19].

To prevent ACV, all the patients with aSAH received routine maintenance treatment with continuous intravenous nimodipine at a dose of 2 mg/h for at least 4 days. Then, the patients took oral nimodipine (360 mg) six times a day for more than 2 weeks.

This study was approved by the Institutional Review Board of Ilsan Paik Hospital, including the review and publishing of information obtained from patient records (IRB no. 2021-10-017-001).

### 2.2. Statistical Analysis

The patients were divided into two groups based on cilostazol medication: the nimodipine-only group (nimo group) and the cilostazol + nimodipine group (cilonimo group). We studied the incidence of ACV and DCI in the cilonimo group compared to the nimo group, including demographic features and radiological findings.

The chi-squared test or Fisher’s exact test was used to compare categorical variables, whereas the *t*-test was used to compare continuous variables. Multivariable logistic regression with stepwise selection was used to identify independent clinical and radiologic risk factors associated with the development of ACV or DCI. A *p*-value < 0.05 was considered statistically significant. All the analyses were performed using the IBM SPSS software 21.0 version (IBM, Armonk, NY, USA).

### 2.3. Hybrid Approach for Feature Analysis and DCI Effect Prediction

To analyze the importance of DCI prediction and the contributing dominant risk factors, we adopted an explainable predictive modeling method and statistical analysis. XAI can be used to visually predict how much factors contribute to the development of DCI in individual patients with SHAP and LIME. However, the above statistical techniques can be used to intuitively confirm the importance of the prognostic factors and the statistical significance of the cilostazol and nimodipine use groups.

#### 2.3.1. XAI

**Figure 1:**
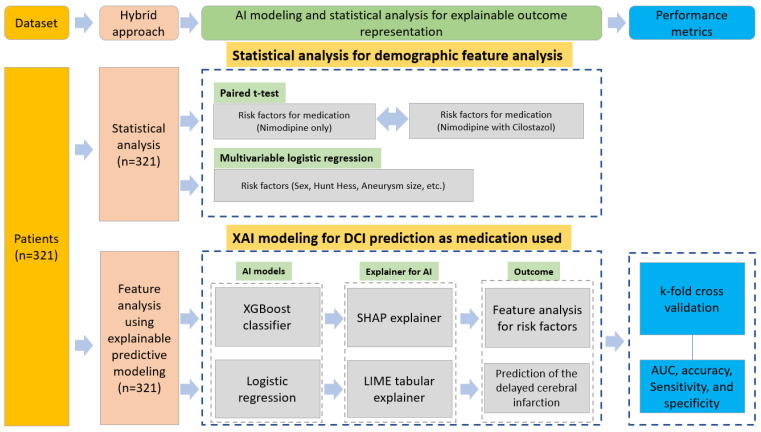
Research diagram for predicting DCI and analyzing risk factor contribution.

The predictive models were built using XGBoost and logistic regression. After applying the AI model, interpretation power was imposed using a model explainer to analyze the contribution of factors to the prediction results [20]. An added procedure for interpreting the prediction results was performed using Shapley additive explanations (SHAP) and local interpretable model-agnostic explanation (LIME) methods, as shown in Figure 1. Global feature analysis of the factors affecting DCI was performed using the XGBoost classifier with SHAP explainer [21,22]. Additionally, we predicted the probability of DCI onset for each patient and performed local feature analysis of the risk factors caused using logistic regression with the LIME explainer. In summary, SHAP was utilized to derive the feature importance for the prognostic factors such as age, aneurysm size, etc., while LIME was employed to make predictions regarding individual prognoses for DCI.

##### SHAP

SHAP was devised by L. Shapley and is a value obtained by probabilistically calculating how the participants in a game contributed to the game result [23]. That is, the SHAP value, *Φ_i_* (*Ν*), for the risk factors for DCI can be used to calculate the ratio of the contribution of a risk factor based on the weight of the contribution of all predictors, as shown in Equation (1):

(1) $$\Phi_{i}\left(N\right)=\frac{1}{\left|N\right|!}\sum_{R}\left(v\left(P_{i}^{R}\cup \left\{i\right\}\right)-v\left(P_{i}^{R}\right)\right)$$

where *N* is the number of players (risk factors), $P_{i}^{R}$ is the set of risk factors, $v\left(P_{i}^{R}\right)$ is the contribution of a set of risk factors, and $v\left(P_{i}^{R}\cup \left\{i\right\}\right)$ is the contribution of a set of risk factors in order and with factor *i*.

In detail, the formula, Equation (1), for SHAP involves computing the difference in predictions with and without each feature in all possible feature combinations. The SHAP value for a particular feature is determined by taking the average variation in predictions when that feature is present or absent in all conceivable combinations with other features.

##### LIME

The explainable model, for instance, *x*, is the model, *g*, that minimizes loss, *L*. It measures how close the prediction and explanation of the original ML model, *f*, are. However, the complexity, Ω(*g*), of the model should be kept low. In this case, *G* is a set of possible explanations, and this is defined as explanation $\overset{\text{\textasciicircum{}}}{g}$ [24]:

(2) $$\overset{\text{\textasciicircum{}}}{g}=\arg\underset{g\in G}{\min}L\left\{f,g,v\left(x\right)\right\}+\Omega \left(g\right)$$

The goal is to discover an interpretable model *g* that minimizes the loss function while adhering to the regularization constraints. LIME accomplishes this by addressing an optimization problem that strikes a balance between preserving robustness to the original model and ensuring the interpretability of the explanations.

In LIME, the importance of each feature in the interpretable model is determined by assessing how much it contributes to the predictions for an individual data instance. LIME is used to explain the prediction made by the AI model for the selected instance by assigning feature importance values. These explanations are then visualized by highlighting the important features, especially for prognostic factors in DCI prediction.

#### 2.3.2. K-Fold Cross-Validation and Programming Environment

K-fold cross-validation is a widely used method in machine learning to evaluate how well a model performs and generalizes. It works by dividing the dataset into k subsets, or folds, and then running several rounds of training and testing using different combinations of these folds. There are several reasons why k-fold cross-validation is advantageous. For reliable performance assessment, unlike relying on a single train-test split, k-fold cross-validation offers a more dependable estimate of a model’s performance. It addresses the influence of data randomness or bias that can occur with a single split. By averaging the outcomes from multiple iterations, k-fold cross-validation delivers a more robust and representative evaluation of the model’s performance [25,26,27].

In summary, k-fold cross-validation plays a crucial role in assessing and comparing models, optimizing hyperparameters, and obtaining trustworthy performance estimates. It facilitates the development of robust and generalizable machine learning models by utilizing the available data effectively and providing deeper insights into the model’s performance characteristics. In this study, we used 5-fold cross-validation.

The area under the curve (AUC), accuracy, sensitivity, and specificity were calculated as the performance metrics (Figure 1).

Python 3.8.3, scikit-learn 0.23.1 for ML, SHAP 0.36.0, and LIME 0.2.0.1 modules were used in the programming environment.

## 3. Results

### 3.1. Statistical Analysis

A total of 321 consecutive patients with aSAH satisfied the study’s inclusion and exclusion criteria.

Table 1 shows the demographic features and risk factors for ACV and DCI according to the cilostazol use of the patients with aSAH who were included in the final analysis. A total of 89 (27.7%) patients received cilostazol combined with nimodipine (the cilonimo group), and 232 (72.3%) received nimodipine only (the nimo group).

The mean size of aneurysms in the cilonimo group was significantly larger than that in the nimo group (6.55 mm versus 5.63 mm; *p* = 0.029). Regarding the treatment method, the cilonimo group included more coiling cases (82%), and the nimo group included more clipping cases (72.8%). ACV and DCI were less frequent in the cilonimo group than in the nimo group (ACV: 13.5% vs. 29.3%; *p* = 0.003; DCI: 7.9% vs. 20.7%; *p* = 0.006).

There were no statistically significant differences in age, sex, GCS, Hunt–Hess grade and Fisher grade, and vascular risk factors between the two groups on admission.

In the multivariate logistic regression analysis of risk factors, cilostazol combined with nimodipine was independently associated with lowering DCI development. Additionally, female sex, age, and aneurysm size showed statistical significance (Table 2).

### 3.2. Global Feature Analysis of the Risk Factors Related to DCI Using XAI

**Figure 2:**
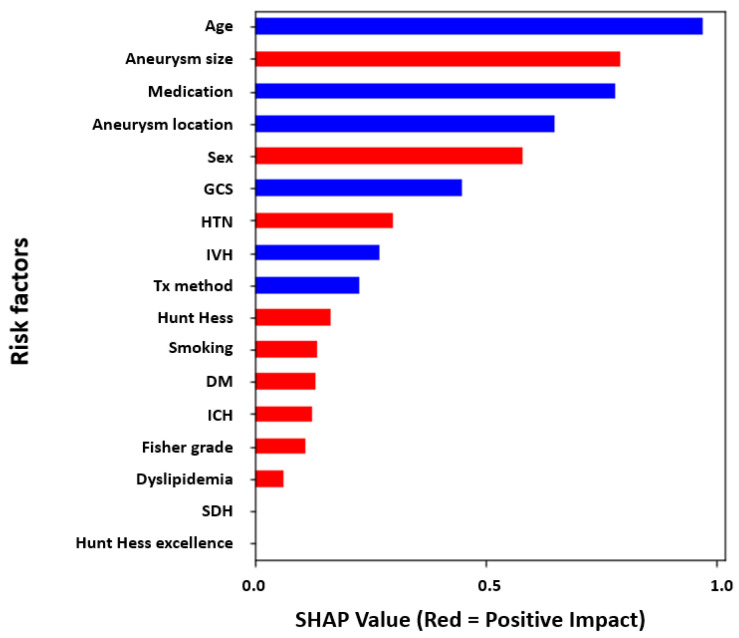
Feature analysis of the risk factors for DCI. Features explained using a global method which is averaged using contributions from all patients using SHAP explainer (ICH: intracerebral hemorrhage, IVH: intraventricular hemorrhage, SDH: subdural hemorrhage, GCS: Glasgow Coma Scale, HTN: hypertension, DM: diabetes mellitus, Tx_method: treatment method (clipping or coiling), Aneurysm_Loc: aneurysm location, HH_Excel: good Hunt–Hess grade (1 and 2) or bad grade (3, 4, and 5), and Aneurysm_Sz: aneurysm size, same as others in Table 1).

Feature importance was analyzed for the importance of the risk factors that could cause DCI in the patients (n = 321). Through the analysis of the retrospective study results, 17.13% of patients experienced DCI, and 82.87% of patients did not experience DCI. The result of analyzing the factors contributing to the DCI is shown in Figure 2. The results showed that the female sex, larger aneurysm size, underlying diseases such as HTN and DM, presence of intracerebral hemorrhage (ICH) and dyslipidemia, and a higher Hunt–Hess grade had a positive impact on DCI (red bars). Conversely, the higher the score of the GCS, the lower the age, within the cilonimo group, the absence of intraventricular hemorrhage (IVH), and the lower the Fisher grade contribute to a lower probability of DCI events (blue bars).

**Figure 3:**
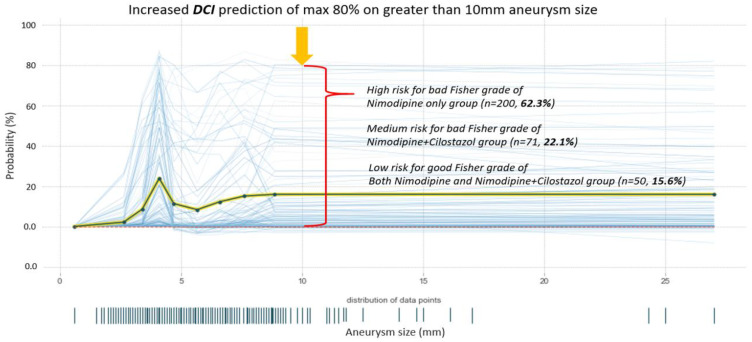
Prediction analysis results of DCI-occurring probability for increased aneurysm size. DCI increased up to 80% chance with an aneurysm size greater than 10 mm and poor Fisher grade (3–4). The high-risk DCI instances were predicted for bad Fisher grade in most of the patients in the nimo group (n = 200, 62.3%). However, medium-risk DCI events were predicted in the cilonimo group (n = 71, 22.1%). Low-risk DCI was predicted in all groups (n = 50, 15.6%).

The prediction analysis results of the DCI-occurring probability for increased aneurysm size are shown in Figure 3. In terms of average age (55.47 years) and aneurysm size (0.6 to 27 mm), it was shown that the incidence of DCI increased up to an 80% chance with an aneurysm size greater than 10 mm and poor Fisher grade (3–4). In terms of prediction instances, the high-risk DCI instances were predicted for bad Fisher grades in most of the patients in the nimo group (n = 200, 62.3%). However, medium-risk DCI events were predicted in the cilonimo group (n = 71, 22.1%). Furthermore, low-risk DCI was predicted in all the groups (n = 50, 15.6%).

### 3.3. Local Feature Analysis of the Risk Factors Related to DCI

**Figure 4:**
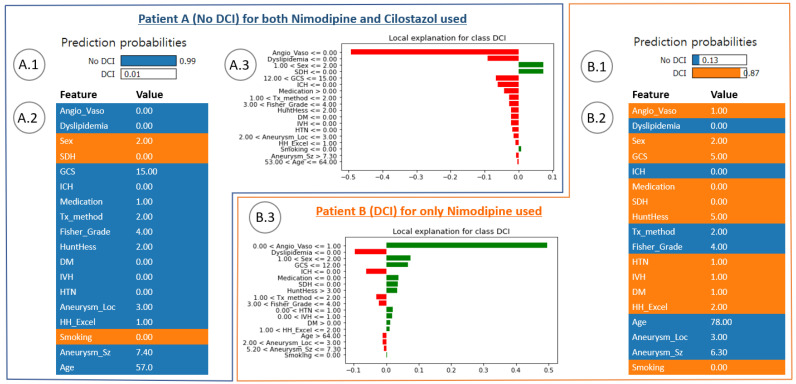
DCI and no-DCI prediction results for specific patient cases of using nimodipine combined with cilostazol (ground truth was no DCI) or nimodipine only. (**A.1**) DCI prediction result for both nimodipine and cilostazol used together. (**A.2**) Risk factors contributing to the feature value. (**A.3**) Local explanation graph for the patient. (**B.1**) DCI prediction result for nimodipine used only. (**B.2**) Contributing risk factors in the feature value. (**B.3**) Local explanation graph for the patient. Angio_Vaso: angiographic vasospasm, Tx_method: treatment method, Aneurysm_Loc: aneurysm location, HH_Excel: good Hunt–Hess grade (1 and 2) or bad grade (3, 4, and 5), and Aneurysm_Sz: aneurysm size, same as others in Table 1.

Figure 4 shows the DCI and no-DCI prediction results for specific patient cases of the cilonimo group (ground truth was no-DCI) and nimo group. Figure 4(A.1) shows the DCI prediction results of using nimodipine combined with cilostazol. Figure 4(A.2) shows the risk factor contributing to the characteristic value. Figure 4(A.3) shows the analysis of the local explanatory graph for the patient. Conversely, Figure 4(B.1) shows the DCI prediction results using nimodipine only. Figure 4(B.2) shows the risk factors contributing to the characteristic value. Figure 4(B.3) shows the local explanatory graph for the patient. These two cases showed a significant difference between the antiplatelet groups. Patient A had no underlying disease (HTN, DM, or smoking history) and had a good Hunt–Hess grade, Fisher grade, and GCS. Because of these factors, the probability of no DCI event occurring was predicted to be 99% for the case of using both nimodipine and cilostazol together. Conversely, patient B had underlying diseases (HTN and DM) and had poor IVH, GCS, Hunt–Hess grade, and Fisher grade. Based on this, the probability of the occurrence of a DCI event was predicted to be 87% for the case of using nimodipine only.

### 3.4. Performance Evaluation Using ML Modeling

**Figure 5:**
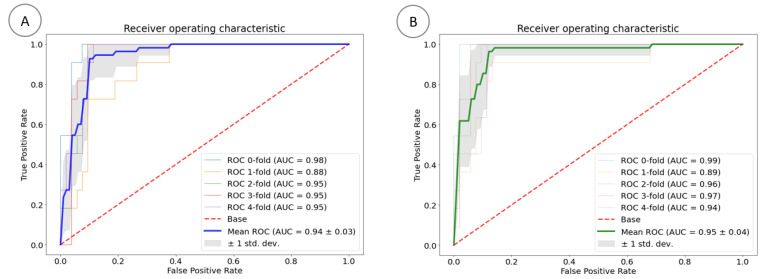
ROC for the XGBoost classifier (**A**) and logistic regression (**B**) models.

The performance of the predictive modeling system was evaluated (Figure 5). The receiver operating characteristics of the XGBoost classifier and logistic regression are shown in Figure 5A,B, respectively. The mean area under the curve (AUC) of the XGBoost classifier and logistic regression models was calculated (0.94 ± 0.03 and 0.95 ± 0.04, respectively). The accuracy, sensitivity, and specificity of the XGBoost and logistic regression were calculated in Table 3.

## 4. Discussion

### 4.1. Conventional Analysis of Cilostazol Effect on DCI in Patients with aSAH

In the literature, there are studies that analyzed the data obtained to confirm the factors predicting a DCI [28,29,30,31]. A young age, aneurysm size, a large volume of SAH, poor neurologic grade on admission, smoking, HTN, and female gender were known as risk factors for vasospasm following aSAH [31,32]. Although there are many studies on patients and disease factors for the occurrence of DCI, DCI still has the most significant impact on the prognosis of patients with aSAH.

Several studies have been published to clarify the pathogenesis of DCI due to ACV. However, no definite cause has been identified. Because of multifactorial reasons, there has not been a single prevention or treatment medication to protect against ACV and DCI. Nimodipine medication for DCI has been used for preventing DCI in many neurosurgical centers. CCBs show strong evidence of preventing DCI in patients with aSAH [33]. Nimodipine is the only medication approved by the U.S. Food and Drug Administration for ACV [10]. Potential mechanisms of the benefits of nimodipine are a neuroprotective cellular effect and the ability to inhibit platelet function by inhibiting thromboxane B_2_ release. However, the cerebrovascular vasodilator effect demonstrated in animal studies has not been demonstrated in a human study [34].

Cilostazol is another option drug studied to prevent ACV [35]. Experimental and small clinical studies have shown good prognostic results in reducing ACV and DCI [36,37,38]. A recent meta-analysis showed that, with the use of cilostazol, the prevalence of symptomatic vasospasm decreased with an odds ratio of 0.35 (95% confidence interval (CI), 0.21–0.59; *p* < 0.0001) [10]. Another meta-analysis by Saber showed that cilostazol was associated with a decreased risk of symptomatic vasospasm (0.31, 95% CI, 0.20–0.48; *p* < 0.001) and DCI (0.32, 95% CI, 0.20–0.52; *p* < 0.001) [39]. Recently, two meta-analyses reported that the cilostazol group significantly reduced DCI incidence. In our study, the cilostazol group showed a marked decrease in DCI, but the odds ratio was about 0.556. We thought that this difference appeared to be due to the control group. Most of the included and analyzed papers in the meta-analysis did not use nimodipine in the control groups at all.

Although many studies have reported that nimodipine and cilostazol can reduce DCI in patients with SAH, there are few reports on whether the combined treatment of both drugs has a better effect in preventing DCI [11,35]. Our data showed that the use of cilostazol combined with nimodipine decreases the incidence of DCI and ACV compared to nimodipine alone. Our study provides implications for future studies that the treatment using a multidrug with two or more drugs may improve the clinical outcome and prognosis more than treatment with no or one drug in patients with aSAH.

### 4.2. Explainable Modeling in Patients with aSAH

There have been several studies on the early prediction of delayed cerebral ischemia in patients with SAH using ML. Tanioka et al. calculated the 95.1% predictive accuracy of delayed cerebral ischemia using a random forest model using early stage clinical data from 95 patients with SAH [17]. Periostin, osteopontin, galectin-3, and the aneurysm location were analyzed in a prognostic factor analysis of delayed cerebral ischemia. However, the importance of each feature has not been confirmed in a delayed cerebral ischemia model that combines the matricellular protein and clinical variables concerning how the importance of each feature affects an individual patient. In other words, it has the limitation of not analyzing explainable predictive outcomes using the SHAP and LIME models. Furthermore, Savarraj et al. obtained an accuracy of a maximum of 0.89 ± 0.03 (95% CI, 0.81–0.94) for 399 patients with DCI using a support vector machine and random forest model [14]. To improve SAH management, the 3-month outcome for the patient was predicted. They focused only on identifying the potential of using machine learning algorithms to improve the prediction of delayed cerebral ischemia and functional outcome after a subarachnoid hemorrhage. That is, individual patient-specific contributions of clinical prognostic factors contributing to DCI were not analyzed using global and local interpretations.

Meanwhile, Ramos et al. used a clinical and baseline CT image dataset from 317 patients with aSAH. To predict delayed cerebral ischemia, they used a logistic regression model to evaluate the prognostic values of predictors [40]. At this time, the AUC for the logistic regression model was 0.63 (95% CI, 0.62–0.63). It was reported that the ML algorithm significantly improved delayed cerebral ischemia predictions in patients with aSAH. Additionally, only local analyses of patients developing delayed cerebral ischemia using LIME were performed, except for global interpretation concerning the prognostic features.

With our unique approach, the contributing risk factors, including sex, age, underlying disease, hemorrhage after aSAH, and a common grading system (Hunt–Hess grade, Fisher grade, and Glasgow coma), were analyzed and interpreted to predict DCI events using ML models with SHAP and LIME for individual cases (instances). The significant distinction is between global and local feature importance. The global method measures take all predictions into account, whereas the local method measures focus on the contribution of features for a specific prediction with respect to patient cases. SHAP takes the absolute value of the average of the local interpretation results, which is called global interpretation [41] (Figure 2). That is, it can be easily used to statistically interpret the overall trend of the data for the prognostic factors contributing to the dependent variable. However, it is difficult to interpret the cases of certain patients. The LIME explainer, however, is a method that calculates the contribution of the independent variable that contributes to the dependent variable in each instance [42] (Figure 4). Therefore, specific patient cases can be inferred in detail [24]. It is possible to provide an integrated interpretation of all the cases and a more detailed interpretation of individual patient cases at the same time by using two explainers for a consistent interpretation of DCI prediction results.

### 4.3. In-Depth Understanding of Global and Local Interpretations of Risk Factors

In Figure 2 and Figure 4, SHAP and LIME explainers were used to analyze global and local feature importance, respectively. In short, it is optimal to sequentially represent the outstanding values of risk factors with the generalizations of risk factors to all patients. Obviously, this is a determination method for the contribution of average impact for all patients, and the results may not reflect individual patients (Figure 2). Thus, to solve these limitations, an alternative method was used for the locally interpretable model-agnostic explanatory LIME analysis method (Figure 4). LIME has a local interpretation function for individual patients, allowing the patient-specific risk factor contribution. This means that the prediction probability of our AI models has to provide more accurate results in each case than the average prediction accuracy.

Meanwhile, using the k-fold cross-validation method involves repeatability issues. The use of the training model under the same conditions may return different results across the five times of five-split training sets. However, further studies to improve the stability of our predictive models have not been conducted. Furthermore, complementary indices such as a variable stability index and a coefficient stability index can be used to evaluate improvement stability [43].

### 4.4. Prediction Analysis of DCI Probability by Aneurysm Size

Another approach of this study was to analyze the prediction of the DCI occurrence probability for enlarged aneurysms (Figure 3). The GCS, Hunt–Hess, and Fisher grades are factors for diagnosing a patient’s current neurological status and symptoms, and, together with age and the size of a cerebral aneurysm, are important factors to consider in the development of DCI (Table 1).

Regarding the average age (55.47 years) and size of the aneurysm (ranging from 0.6 to 27 mm), the findings demonstrated that the likelihood of DCI occurrence increased significantly, reaching up to an 80% chance in cases where the aneurysm size exceeded 10 mm, and the Fisher grade was poor (graded as 3–4). In terms of predictive analysis, the study identified a high-risk category for DCI in the majority of patients within the nimo group (n = 200, 62.3%), specifically associated with a poor Fisher grade. Conversely, the cilonimo group (n = 71, 22.1%) exhibited medium-risk predictions for DCI events. Additionally, all the groups included cases where low-risk DCI predictions were made (n = 50, 15.6%).

### 4.5. Hyperparameter Tuning within XAI and the Limitation of this Study

Hyperparameter tuning within XAI involves optimizing the hyperparameters of a machine learning model specifically used for explainability purposes [44]. Hyperparameters, unlike trainable parameters, are predetermined by the user before model training commences. In XAI, the process of hyperparameter tuning plays a crucial role in ensuring that the chosen explainability techniques or algorithms are suitably configured for the given task and dataset [25,26,45,46]. The effectiveness and interpretability of the model’s explanations heavily rely on these hyperparameters. Hyperparameter tuning typically entails a systematic exploration of various combinations of hyperparameter values, with the aim of finding the optimal configuration that maximizes the desired outcome. Techniques such as grid search, random search, or more advanced optimization algorithms such as Bayesian optimization can be employed for this purpose. For instance, in a model-agnostic XAI method such as LIME used in this study, hyperparameters such as the number of selected features or the kernel width significantly impact the quality and faithfulness of the generated explanations. Fine-tuning these hyperparameters enables researchers or practitioners to strike a balance between the explanations’ interpretability and their accuracy in representing the underlying model’s behavior. Wu et al. studied the optimization of gcForest (multi-grained cascade forest), one of the decision tree ensemble methods, and published the results [46]. Before optimization, the prediction accuracy was 85.68%, but, after optimization, the prediction accuracy increased to 87.05%.

K-fold cross-validation is beneficial when it comes to comparing various models or fine-tuning hyperparameters. It enables the assessment of different models or parameter configurations across multiple validation sets. This capability leads to improved decision making in terms of model selection and hyperparameter tuning, as it is based on more comprehensive and consistent performance evaluations [25,26].

In future research, we will be able to enhance the clarity, relevance, and reliability of the resulting explanations. Thus, the overall interpretability and trustworthiness of AI systems can be improved, and the lack of datasets can be supplemented through multicenter clinical trials.

## 5. Conclusions

Cilostazol treatment combined with nimodipine could decrease the prevalence of ACV and DCI in patients with aSAH, according to both XAI and statistical analysis.

First, the feature importance analysis using SHAP showed that sex, a larger aneurysm size, underlying diseases such as HTN and DM, the presence of ICH and dyslipidemia, and a higher Hunt–Hess grade had a significant impact on DCI. In addition, individual predictions for patient outcomes using LIME indicated that the probability of a DCI event occurring was predicted to be 87% for the case of a patient who had an underlying disease, IVH, and a poor GCS. Second, the mean size of aneurysms in the cilonimo group was significantly larger than that in the nimo group (6.55 mm versus 5.63 mm; *p* = 0.029). Also, the cilonimo group included more coiling cases (82%), and the nimo group included more clipping cases (72.8%). ACV and DCI were less frequent in the cilonimo group than in the nimo group (ACV: 13.5% vs. 29.3%; *p* = 0.003; DCI: 7.9% vs. 20.7%; *p* = 0.006) in the statistical analysis.

There were no statistically significant differences in age, sex, GCS, Hunt–Hess grade and Fisher grade, and vascular risk factors between the two groups on admission.

Although there is a possibility of increasing the prediction accuracy through an optimization process such as additional hyperparameter tuning, we tried to demonstrate explainable predictive modeling by visually analyzing the importance of prognostic factors and the contribution of individual patients to the occurrence of DCI. Further studies involving multicenter trials and improving the stability of our prediction models using global and local interpretations are needed to strengthen our results.

## Figures and Tables

**Table 1:** Patients’ demographics and dataset characteristics for the predictive modeling.

Characteristics	Nimodipine Only (n = 232)	Cilostazol and Nimodipine (n = 89)	*p*-Value	Data Type	Value
Age, y, mean (SD)	55.47 (12.756)	55.37 (14.150)	0.952	Float	21–94
Sex, female, n (%)	147 (63.4)	51 (57.3)	0.318	Binary	1: male; 2: female
GCS on admission, mean (SD)	12.81 (3.248)	13.25 (3.185)	0.279	Integer	1–15
Aneurysm size, mm, mean (SD)	5.63 (3.016)	6.55 (4.123)	0.029	Float	0.6–27
Hunt–Hess grade	N/A	N/A	0.142	Integer	1–5
1	12	1			
2	115	57			
3	67	20			
4	33	9			
5	5	2			
Fisher grade	N/A	N/A	0.068	Integer	1–4
1	14	6			
2	18	12			
3	103	26			
4	97	45			
Location	N/A	N/A	0.007	Integer	1: ACA; 2: MCA; 3: ICA; 4: VA or BA
ACA	86	40			
MCA	60	10			
ICA	74	28			
VA or BA	12	11			
HTN, n (%)	95 (40.9)	35 (39.3)	0.791	Binary	0 or 1
DM, n (%)	25 (10.8)	13 (14.6)	0.342	Binary	0 or 1
Hyperlipidemia, n (%)	17 (7.3)	12 (13.5)	0.085	Binary	0 or 1
Smoking, n (%)	76 (32.8)	37 (41.6)	0.139	Binary	0 or 1
Clip/coil, n (%)	169 (72.8)/63 (27.2)	16 (18.0)/73 (82.0)	0	Binary	0 or 1
ACV	68 (29.3)	12 (13.5)	0.003	Binary	0 or 1
DCI	48 (20.7)	7 (7.9)	0.006	Binary	0 or 1

Note: GCS: Glasgow coma scale; ACA: anterior cerebral artery; MCA: middle cerebral artery; ICA: internal carotid artery; VA: vertebral artery; BA: basilar artery; HTN: hypertension; DM: diabetes mellitus; ACV: angiographic cerebral vasospasm; DCI: delayed cerebral infarction; N/A: not applicable.

**Table 2:** Multivariate analysis of risk factors for DCI development.

Variables	OR	95% CI	*p*-Value
Cilostazol with nimodipine	0.556	0.351–0.879	0.012
Female sex	3.713	1.683–8.191	0.001
Age	0.972	0.946–0.999	0.042
Aneurysm size	1.106	1.008–1.214	0.034
Treatment method	1.1	0.483–2.502	0.821

**Table 3:** Performance metric of the predictive models.

Models	Accuracy	Sensitivity	Specificity
XGBoost	0.91	0.70	0.95
Logistic regression	0.92	0.80	0.95

## Data Availability

Not applicable.
